# No evidence of spherical microplastics (10–300 μm) translocation in adult rainbow trout (*Oncorhynchus mykiss*) after a two-week dietary exposure

**DOI:** 10.1371/journal.pone.0239128

**Published:** 2020-09-25

**Authors:** Joel Kim, David G. Poirier, Paul A. Helm, Malak Bayoumi, Chelsea M. Rochman

**Affiliations:** 1 Department of Ecology and Evolutionary Biology, University of Toronto, Toronto, Ontario, Canada; 2 Aquatic Toxicology Unit, Ontario Ministry of the Environment, Conservation and Parks, Etobicoke, Ontario, Canada; 3 Environmental Monitoring and Reporting Branch, Ontario Ministry of the Environment, Conservation and Parks, Etobicoke, Ontario, Canada; VIT University, INDIA

## Abstract

The consumption of fish contaminated with microplastics is often cited as a pathway for human exposure. However, because their guts are generally removed before consumption, exposure may be low compared to other routes such as shellfish, drinking water and dust. Still, microplastics have been found to translocate from the gut to other tissues, making exposure from eating fish fillets or other seafood products a potential concern. To better understand fish as an exposure route for microplastics in humans, we tested hypotheses about whether translocation occurs and if efficiency of translocation is dependent on particle size. We investigated the amount and distribution of fluorescent polyethylene microspheres (10–300 μm) in the gut, liver, fillets and gonads of adult rainbow trout after a two-week dietary exposure. Fish were fed food pellets dosed with up to ~9,800 microspheres per gram of food. Total exposures over the entire experiment ranged from ~80,000–850,000 microspheres per fish. We did not find any particles in the fillets, liver, or gonads of any fish, suggesting that translocation of spherical microplastics of this size range does not occur in adult rainbow trout. The quantity of microplastics found in the gut was also low or absent after a 24-hour depuration period, indicating effective excretion in this laboratory population. This research suggests that the consumption of fish fillets may not be a significant exposure pathway for microspheres >10 μm in size to contaminate humans. Future studies should test for different sizes, morphologies and species to further our understanding.

## Introduction

The combination of increasing plastic production, inefficient waste management and its recalcitrance has allowed microplastics to accumulate in the environment [1]. Microplastics incorporate a diverse array of polymers, sizes, densities, shapes and colors [2]. Although there is no consensus on the definition of a microplastic, <5 mm (NOAA) and 1 to <1000 μm have been proposed and widely adopted [3]. Today, microplastics are a truly ubiquitous anthropogenic contaminant which have been reported in a diverse range of habitats such as Arctic waters [3] and ice [4], tropical estuaries [5] and deep–sea sediments [6]. Consequently, over 220 different species have been found to ingest plastic debris *in natura*, including those of commercial importance for fisheries and aquaculture [7]. Although there remain many uncertainties regarding microplastics and their potential to cause harm to humans, they have received continued attention as a threat to food safety and security [8].

Several studies have quantified microplastics in the gut contents of animals commonly consumed as seafood, but their significance as a source of microplastics to the human diet is dependent on how people consume them. Seafood consumed whole (e.g. mussels, smaller fish) are expected to deliver a greater abundance of microplastics to humans per organism relative to those whose guts are removed or discarded [9]. Thus, human exposure is not expected in the latter. However, the fate of ingested plastic particles within organisms is unknown and some researchers have suggested microplastics may translocate to other tissues.

The translocation of microplastics from the gut to other parts of an organism’s body has been documented in both aquatic and terrestrial organisms, such as 0.5 μm particles in the circulatory system of blue mussels [10], 124–438 μm particles in the livers of anchovies (*Engraulis encrasicolus*) [11] and 5 and 20 μm particles in the livers and kidneys of mice [12]. Thus, it is plausible that microplastics are present in the tissues of seafood typically consumed by humans. Given the level of seafood consumption worldwide, it is important to consider translocation in regard to human exposure. In addition, considering the occurrence of translocation is important to fully understand whether microplastics bioaccumulate and biomagnify in food webs like other contaminants.

Microplastics have been shown to transfer across trophic levels. Shore crabs (*Carcinus maenas*) fed blue mussels (*Mytilus edulis*) exposed to 0.5 μm fluorescent polystyrene microspheres were found to contain these particles in the hemolymph and tissues of the crab [13]. Microplastics have been found to transfer from Atlantic mackerel (*Scomber scombrus*) to captive grey seals (*Halichoerus grypus*) [14]. Translocated particles were also shown to remain in the hemolymph of mussels for over 48 days, providing evidence of retention [10]. As a result, concerns have been raised about whether bioaccumulation can occur and whether there is biomagnification or trophic dilution at higher levels of the food web [15]. Although there is copious evidence that microplastics contaminate organisms at all trophic levels, their fate within an organism and how they move through food webs is less clear.

Here, our objective was to determine whether translocation of spherical microplastics ranging from 10 μm–300 μm in size occurs in a commercially important predatory fish. We experimentally measured whether commercially available fluorescent polyethylene (PE) microspheres would translocate from the gut to other organs and fillets of adult rainbow trout (*Oncorhynchus mykiss*). After a 14–day dietary exposure, we quantified the amount and size distribution of microplastics in the gut, liver, fillets and gonads to test the prediction that microplastics could translocate and that smaller microplastics translocate more readily than larger microplastics.

## Materials and methods

### Fish and husbandry

This study was carried out in strict accordance with the recommendations in the Ontario Animals for Research Act and were in agreement with local animal law. An animal utilization proposal detailing the care, maintenance, handling and euthanasia of fish, was approved by the Ministry of the Environment, Conservation and Parks (MECP) Animal Care Committee veterinarian. Rainbow trout were used since they are a native species to North America, of commercial importance and used worldwide as a standard for freshwater toxicity testing [16]. Rainbow trout were received as eyed eggs (lot # RBT–15–06) from Rainbow Springs Hatchery (Thamesford, Ontario) on August 31, 2015 and were reared in the Aquatic Toxicology Laboratory at the MECP in Etobicoke, Ontario. Fish were fed BioTrout 6 mm fish chow from Bio-Oregon (Vancouver, British Columbia). Culturing conditions were as follows: the light cycle was 16 ±1 h light: 8 ±1 h dark (100–500 lux) and fish were kept at 15 ± 2°C. Water quality (i.e. ammonia, pH, dissolved oxygen and conductivity) was monitored daily over the 14–day exposure period and were within the expected range (pH = 7.7 ± 0.71; DO = 9.5 ± 0.17 ppm; conductivity = 288.21 ± 0.55 μS/cm). Ammonia concentrations were measured once daily using Ammonia (Nitrogen) Test Strips, 0–6.0 mg/L purchased from Hach (Loveland, Colarado) prior to each water change when levels were highest. At most, ammonia levels reached two ppm (1.26 ± 0.71 ppm). For more information about water quality, please see S1 Table. Fish did not show overt signs of stress such as darkened pigmentation, sharking or excess mucous production.

For experimentation, fifteen fish were randomly selected from a central culture tank at the MECP and transferred to individual 20 L buckets lined with food grade PE bags. Vigorous aeration was supplied through silica glass air stones. At the time of exposure, fish were roughly three years old, measured 30–35 cm in length and weighed between 400–600 g. Of the 15 fish, seven were male and seven were female (Table 1). The sex for one individual could not be determined. All fish were left to acclimate for 24 hours prior to the 14–day dietary exposure and fed microplastic–free trout chow during acclimation. At the end of the exposure, fish were sacrificed with a blow to the head followed by severance of the cervical spine before any tissue samples were taken. All efforts were made to minimize suffering.

**Table 1:** Summary of fish. Fish total length (TL), weight (g), sex and total quantities of fluorescent PE microspheres from all size ranges found in the gut contents of individual fish after a 24-hour depuration period.

Treatment	Replicate	TL (cm)	Weight (g)	Sex	Total # in gut (all sizes)	Average # in gut ± std. dev.
High	1	32	570	F	1	31 ± 56
	2	32	498	M	0	
	3	31	445	M	0	
	4	31	402	F	143	
	5	32	504	Unknown	11	
Low	1	32	592	F	0	11 ± 20
	2	34	567	M	51	
	3	30	580	M	1	
	4	32	583	M	4	
	5	30	430	F	0	
Control	1	34	614	M	0	0
	2	35	509	M	0	
	3	32	566	F	0	
	4	34	508	F	0	
	5	33	572	F	0	

### Microplastics

**Fig 1:**
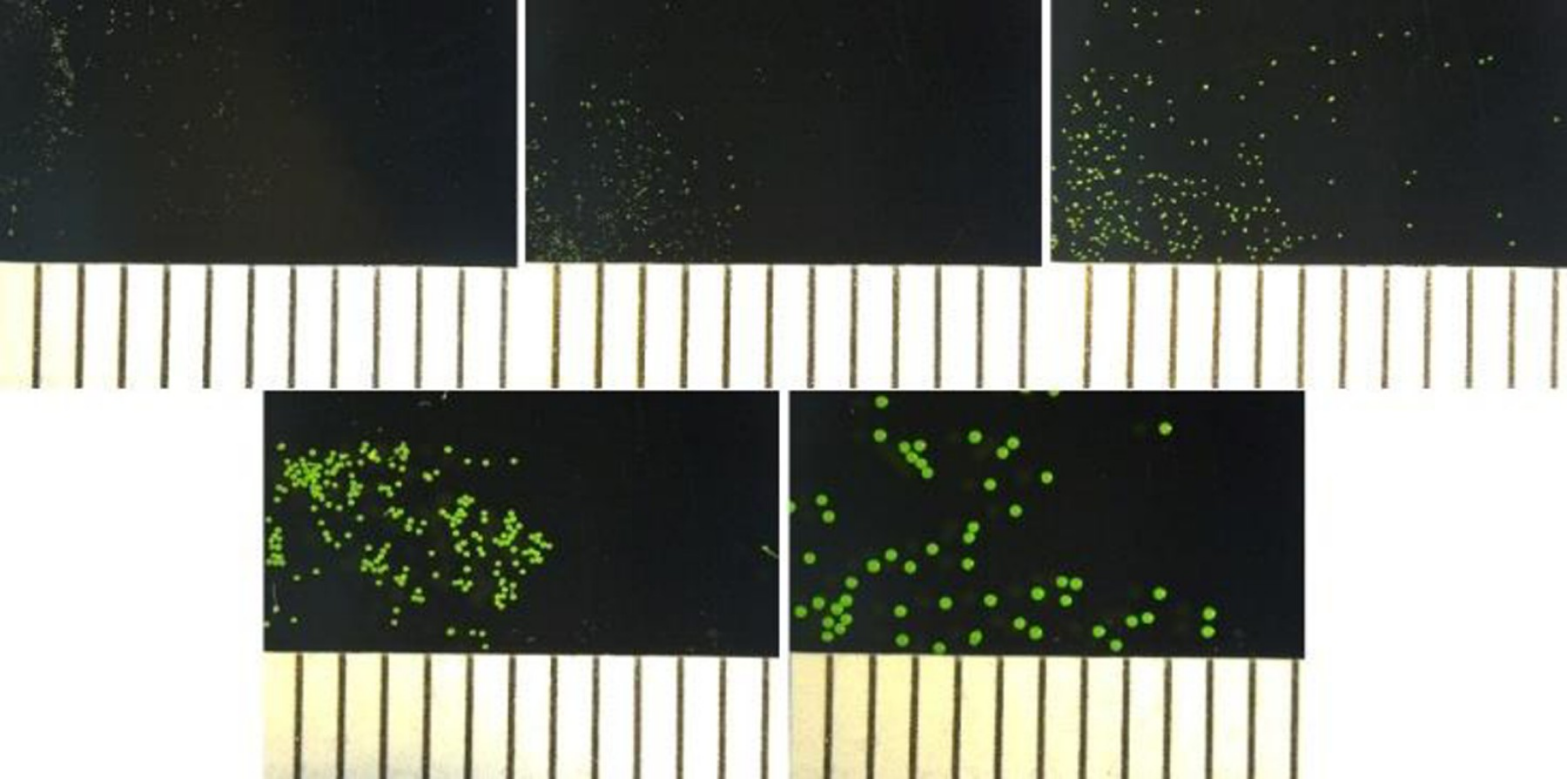
Images of fluorescent PE microspheres. Microplastics used in dietary exposure to *O*. *mykiss*. A) 10–20 μm; B) 27–32 μm; C) 63–75 μm; D) 125–150 μm; E) 250–300 μm. Scale in 1 mm increments.

Fluorescent green PE microspheres with a density of 1.00 g/cm^3^ were purchased from Cospheric (Santa Barbara, California) in five different size ranges: A) 10–20 μm; B) 27–32 μm; C) 63–75 μm; D) 125–150 μm; and E) 250–300 μm (Fig 1). A data sheet estimating the quantity of microspheres per unit mass was provided by Cospheric and used to determine the expected particle count used for experimentation.

### Preparation of diets

To make a “low” and a “high” microplastic diet, BioTrout 6 mm fish chow from Bio-Oregon (Vancouver, British Columbia) was ground using a commercial blender. A nominal quantity of fluorescent beads at different sizes, roughly a total of 700,000 (“low” concentration) and 4,900,000 (“high” concentration) microspheres of each size range were mixed into two separate metal containers with 500 grams of fish food each. The “low” concentration was informed by Jovanović et al. (2018), where 0.3% of total feed was considered environmentally relevant [17]. The “high” concentration was simply seven times the “low” concentration and the highest concentration that was not too resource intensive. In general, this experiment was not designed to be environmentally relevant, but to assess translocation. We used relatively large concentrations in an attempt to ensure translocation would occur, if possible, and chose fluorescent green beads to minimize external contamination and identification issues.

The expected concentrations for the diets were ~1,400 microspheres per gram of food for the “low” concentration and ~9,800 microspheres per gram of food for the high concentration (S2 Table). After adding 250 mL of water, these mixtures were homogenized using a hand mixer, extruded out of a stainless-steel pelletizer and cut into pieces the fish can consume. The diet was oven-dried at 60°C for 24 hours and then frozen at –20°C until the exposure period.

### Homogeneity of diets

To confirm homogeneity of microplastics in the “low” and “high” treatment diets, five grams of diet (n = 5 samples per treatment diet) were digested using an optimized KOH digestion [18], sieved based on size and quantified using a FlowCam VS purchased from Fluid Imaging Technologies, Inc. (Scarborough, Maine). Particles were added to a 1:1 Decon Contrad 70 Liquid Detergent from Thermo Fisher Scientific (Ottawa, Ontario) and water mixture to prevent clumping of particles. Using the FlowCam VS, particles were quantified in each sample and the percent relative standard deviation (%RSD) was calculated between samples from the same treatment for the 125–150 μm and 250–300 μm particles (S1 Fig). Each sample was run on the instrument one time and the %RSD was calculated for each set of five samples. %RSD between samples within the same size fraction was 11.8% for the 125–150 μm size fraction and 16.9% for the 250–300 μm size fraction. To determine the precision of the FlowCam VS, one random five-gram sample was taken from each diet and run on the instrument five times. %RSD between sample runs was 2.86% for the 125–150 μm size fraction and 13.5% for the 250–300 μm size fraction.

We assumed the diets were homogenous when the %RSD between samples within a treatment diet and size range was below 20%. Due to limit of quantification issues with the smaller size fractions (<125 μm) on the FlowCam VS, methodological challenges and based on the consistency of the two size fractions measured, we applied the assumption that the microspheres were homogenous to the other size fractions in the diets.

### Microplastic exposure

We conducted a 14–day dietary exposure with three treatments (n = 5). Treatments were randomly assigned to the fifteen buckets, each containing one individual fish. The treatments included: 1) a “low” exposure consisting of ~1,400 microspheres per gram of fish food with roughly equal quantities of differently–sized particles; 2) a “high” exposure consisting of ~9,800 microspheres per gram of fish food with roughly equal quantities of differently–sized particles; and 3) a negative control, where the fish were fed microplastic–free fish chow. All fish were fed 1% body weight per day starting on August 28^th^, 2018 based on their initial masses. During feeding, each fish was observed until it was verified that the fish consumed all food pellets. Fish were transferred to fresh water daily with a soft polycotton net.

### Sample digestion and quantification of particles

Twenty–four hours after the end of the feeding trial, fish were euthanized. They were then individually placed in freezer bags and transferred to a –20°C freezer until dissection. Prior to dissection, the length of each fish was measured and ranged from 30–35 cm in length. When the fish were dissected, their entire gut (including stomach, pyloric ceca and intestines), liver, fillets and gonads were individually sampled, stored in Whirl–Pak bags and weighed. Sex was determined upon dissection by inspecting the gonads. To avoid contamination, fish were carefully dissected in increasing order of microplastic exposure and the gut was dissected last. All tools, including the cutting board used during dissection, were thoroughly rinsed between each fish to avoid cross contamination.

For digestions, all samples were individually placed in PE containers and treated with five times the sample volume of 10% KOH (w/v) for 24 hours in a 60°C oven, an effective protocol for the extraction of microplastics from seafood [18]. Recovery test results from developmental work using 125–150 μm fluorescent PE microspheres indicated recoveries of 88 ± 11.66% for rainbow trout fillets and 86 ± 10.20% for fish oil (S3 Table). As previously mentioned, recovery tests could not be conducted on the smaller particle sizes. However, the use of 10% KOH to extract microplastics from fish has been found to have ~100% recovery rates and had limited impacts on the integrity of PE for sizes ranges <300 μm and <80 μm [19]. Although we were unable to do our own recovery tests for particles <125 μm, we feel confident in our ability to recover small microspheres down to 10 μm because we were able to see them after digestion in both the diet and the guts of the fish.

To assist in filtering, samples were briefly heated to 60°C and filtered through a vacuum unit containing a 10 μm MilliporeSigma polycarbonate filter purchased from Thermo Fisher Scientific (Ottawa, Ontario) with a copious amount of detergent and water. Samples containing persistent fatty residues were further diluted with a 1:1 mixture of detergent and water prior to filtering. All tools and containers that came into contact with samples were rinsed and visually inspected under a microscope, including the filter unit, to ensure that no microspheres were lost.

To quantify microplastics in all samples, the filters were examined visually under a stereomicroscope capable of up to 80X magnification (OMAXmicroscope.com). To verify that all of the fluorescent microspheres were accounted for, filters were further inspected under a fluorescence microscope.

## Results and discussion

To better understand fish as an exposure route for microplastics to reach humans, we investigated translocation of microplastics in adult rainbow trout, a commercially important fish species. During experimental trials, all fish were observed to consume food pellets. No mortalities occurred nor were there any signs of distress prior to euthanasia. No microplastics were observed outside of the gut, i.e., in the liver, fillets, or gonads, in any fish from all treatments. Thus, our results showed that 10–300 μm virgin PE microspheres did not translocate from the gut to other parts of the body in adult rainbow trout over the exposure period. Although some researchers have found that translocation can occur in different species of fish, such as to the edible tissues of fish and crustaceans [20] and gilt-head seabream (*Sparus aurata*) [17], others have found that microplastics were constrained to the gut [21]. Our observed results, and the results of others, may be based on the size of the particles used [10], but may also be a result of differences in other factors such as the shape of microplastics, the exposure concentration, organism, their life stage and initial contamination. Further work teasing how these factors affect translocation is warranted.

**Fig 2:**
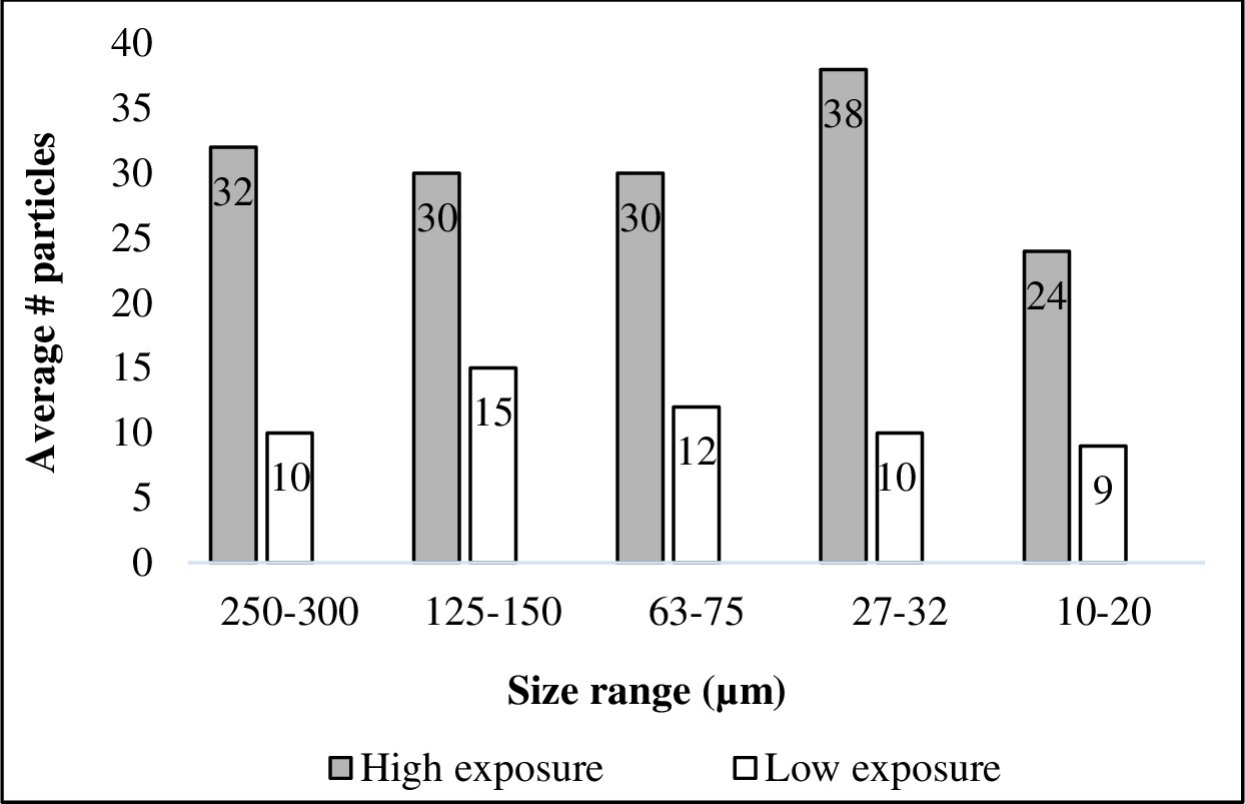
Microplastics found in gut of adult rainbow trout. Total quantity of microplastics in each size fraction found in the gut across all exposed fish in each treatment group (high = grey; low = white). No particles were observed in any control fish.

Here, we saw some retention of microplastics in the gut of the fish 24 hours post-feeding. Microspheres were present in the gut of six of the ten fish that were exposed (three fish each from the “low” and “high” treatments). The quantities found varied considerably, with 10–143 particles found in the high treatment fish, and 0–51 particles in the low treatment fish. Particles were absent altogether in two fish from each of the high and low treatments, and, as expected, were completely absent from the controls (Table 1). This high variability may be due to our low replication (n = 5 per treatment diet). Variability at lower size fractions due to methodological issues cannot be ruled out as our recovery tests were conducted at size fractions >125 μm. Of the fish that contained microspheres in their gut at the end of this experiment, there tended to be a greater amount present in the “high” exposure treatment. However, there appears to be no distinguishable trend to merit a statistical evaluation of differences between treatments. In addition, there was no evidence of retention by particle size. Particles from each size fraction were found and sizes were distributed roughly equally within fish exposed to each treatment diet (Fig 2). This result also supports our assumption that all size fractions were homogenously distributed across each treatment diet.

Although an objective of our research was to determine the size distribution of translocated particles, our results showed that translocation of PE microspheres between 10–300 μm in size does not occur in adult rainbow trout. In other studies, smaller microplastics have been shown to be more likely to translocate than larger microplastics. For example, 3 μm and 9.6 μm fluorescent polystyrene microspheres have been shown to translocate to the circulatory system in blue mussels (*M*. *edulis*), with the smaller 3 μm microspheres found in higher abundance [10]. Moreover, 5 μm, but not 20 μm, polystyrene microplastics were found to translocate to the liver of zebrafish [22]. Finally, polystyrene particles <1 μm (0.25 ± 0.06 μm) have been shown to translocate into human cells [23]. Based on the literature, particle size appears to be an important factor determining translocation [10,23]. Unfortunately, our experimental design may not have been able to capture this accurately for rainbow trout.

The evidence suggesting translocation can occur, begs the question about whether bioaccumulation and biomagnification of microplastics in a food web is possible. Such a question is important ecologically as this may impact whether microplastics can have indirect effects in addition to direct effects. In addition, it is also important to apex predators and humans, as both tend to feed from the top of the food chain. To date, results from studies investigating trophic transfer have observed a lack of evidence for bioaccumulation and biomagnification. To the best of our knowledge, no study has directly tested bioaccumulation–unless translocation is considered bioaccumulation. As for whether or not magnification or dilution occurs, some studies have observed dilution. Crabs fed mussels exposed to roughly 411 million 0.5 μm fluorescent polystyrene microspheres were found to contain roughly 163,000 particles in the crabs, or 0.04% of the total exposure amount [13]. Trophic dilution of microplastics has also been found to occur in the edible muscle of seafood, with organisms from higher trophic levels containing fewer microplastics (mostly fragments <50 μm and fibers 50–8000 μm) than those from lower trophic levels [24]. In this study, microplastics that were consumed by fish were generally excreted, suggesting that spherical microplastic particles >10 μm are unlikely to bioaccumulate and biomagnify in fish.

The presence of microplastics in the gut of fish does not necessarily provide direct evidence of human exposure since this organ is often removed prior to consumption. Thus, exposure resulting from the consumption of fish will be decreased upon removing the gut. Seafood species that are consumed whole (e.g. small or juvenile fish, some crustaceans and shellfish) may pose a greater source of microplastics to humans [25]. However, even at the highest estimated scenario for heavy seafood consumers, the percentage of microplastics consumed in the total diet of an average adult is estimated to be <0.1% by mass [7]. In addition, although 95% of popular commercial bivalves purchased from three major cities in South Korea were found to be contaminated with microplastics, they were estimated to contribute only 212 microplastics per person annually [26]. As South Korea had the highest per–capita seafood consumption of 58.4 kg annually between 2013–2015 (http://faostat.fao.org/) [26], microplastics resulting from seafood consumption are likely even lower for other countries.

Apart from seafood, microplastics have been reported in bottled water [27,28], salt [29–31], beer [32,33] and honey [34], which may provide dietary sources greater than seafood. Exposure from airborne fibers that humans consume inadvertently as a result of it landing on our food may also be a larger source to humans relative to seafood. For example, microplastic ingestion due to consumption of mussels, which are consumed whole and likely pose the “worst case” scenario, were predicted to range between 123–4,260 particles/year/capita; by comparison, plastic ingestion via dust fallout in a household during a meal was predicted to be 13,731–68,415 particles/year/capita [35]. A growing body of literature has begun to show that seafood is a relatively low exposure route of microplastics to humans relative to other routes such as airborne dust and drinking water [36]. Incidental sample contamination by airborne fibers is also a concern when strict control measures are not employed [37,38]. Although microplastics are omnipresent and there is no doubt we are exposed to them via various routes, the potential implications to human health still remain poorly understood.

Overall, the current state of evidence, including our results, imply that the translocation of microplastics may be context dependent and may differ by size, shape and type of the plastic particles, as well as the taxa and life stage of an organism. For example, the physiology of the stomach lining, the size of the digestive tract in different species, or the life stage, in relation to the particle size, may influence the fate of microplastics in an organism. Further research is thus needed to determine whether there are factors such as shape and size that favor translocation in organisms and how this varies across taxa. When translocation does occur, it is also plausible that associated chemicals can transfer to an organism and future research should attempt to quantify chemical transfer via translocation as well. Overall, there is a need for a better understanding of the different exposure levels and pathways for microplastics in animal food webs and in humans. This is particularly the case in order to conduct a risk assessment for ecological and human health.

## Supporting information

S1 Fig Microplastic homogeneity in fish feed. Each bar is the amount of particles/mL in one digested replicate of diet (n = 5) for size ranges 250–300 μm and 125–150 μm.
(TIF)

S1 Table Water quality parameters. (TIF)

S2 Table Mass and quantity of particles added to diet. Microspheres were added to 500 grams of fish food for each treatment. Low treatment corresponds to 1,400 microspheres per gram of fish food and high treatment corresponds to 9,800 particles per gram of fish food.
(TIF)

S3 Table Recoveries for 125–150 μm PE microspheres. Recovery test results from developmental work using 125–150 μm fluorescent PE microspheres. These tests underwent the same protocol as samples.
(TIF)

S1 Raw data Summary of fish. Particle distribution by size found in the gut contents of individual fish after a 24-hour depuration period.
(TIF)
