# Supplementary figures and images for: No evidence of spherical microplastics (10–300 μm) translocation in adult rainbow trout (Oncorhynchus mykiss) after a two-week dietary exposure
Source: PLoS One. 2020 Sep 25;15(9):e0239128. doi: 10.1371/journal.pone.0239128 (PMC7518578; doi:10.1371/journal.pone.0239128)

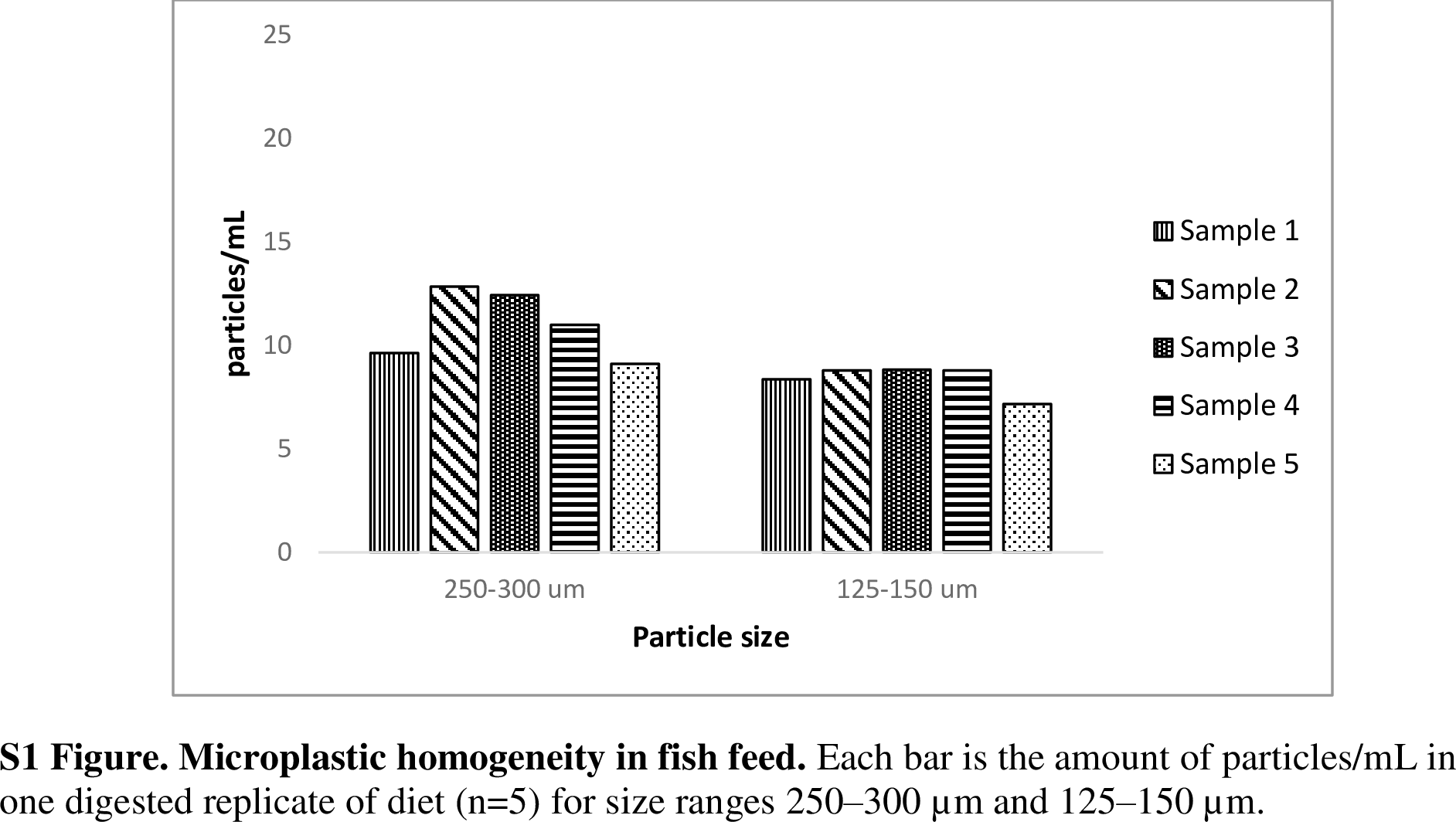

Supplement: S1 Fig — Each bar is the amount of particles/mL in one digested replicate of diet (n = 5) for size ranges 250–300 μm and 125–150 μm. (TIF) [file pone.0239128.s001.tif]

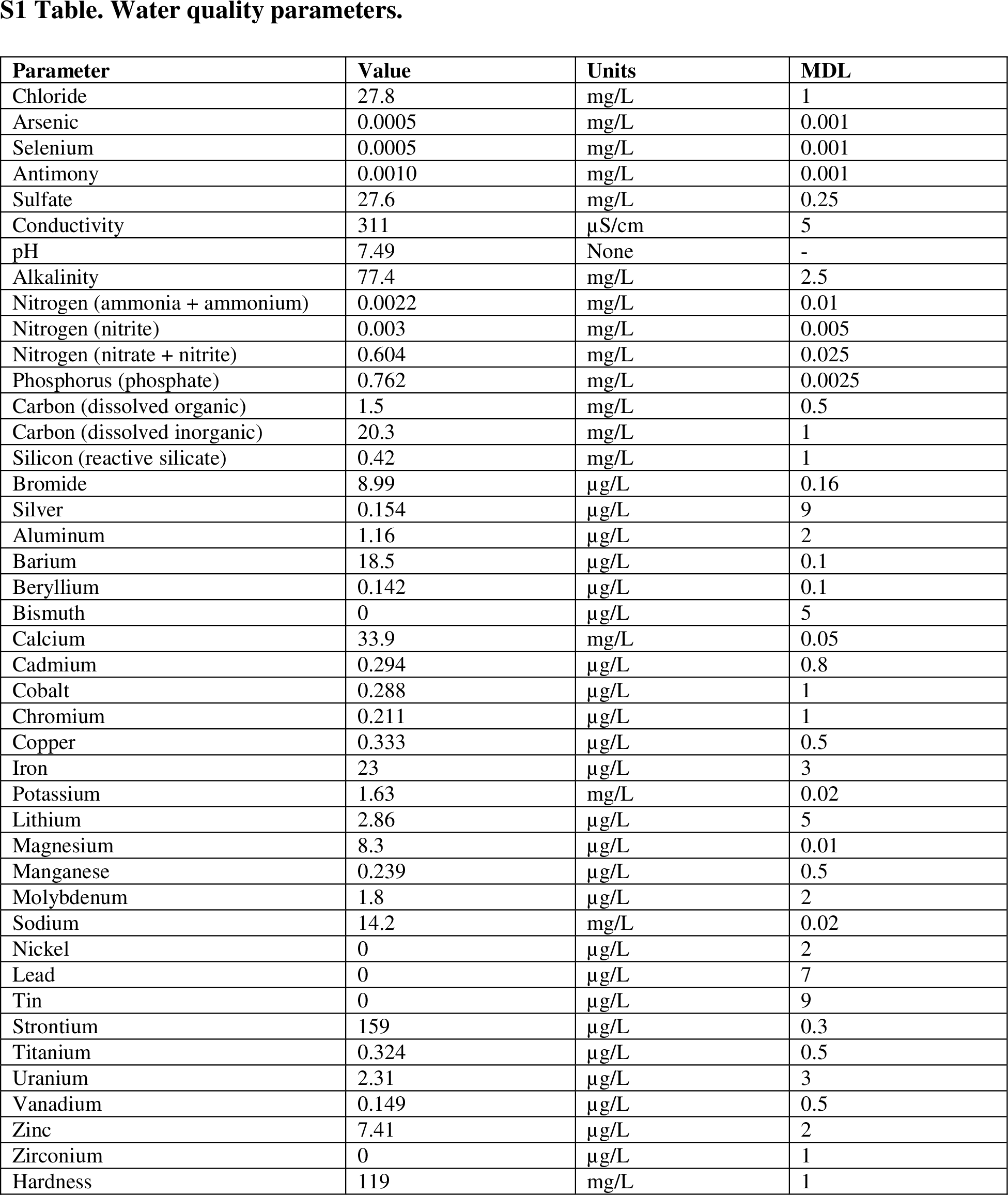

Supplement: S1 Table — (TIF) [file pone.0239128.s002.tif]

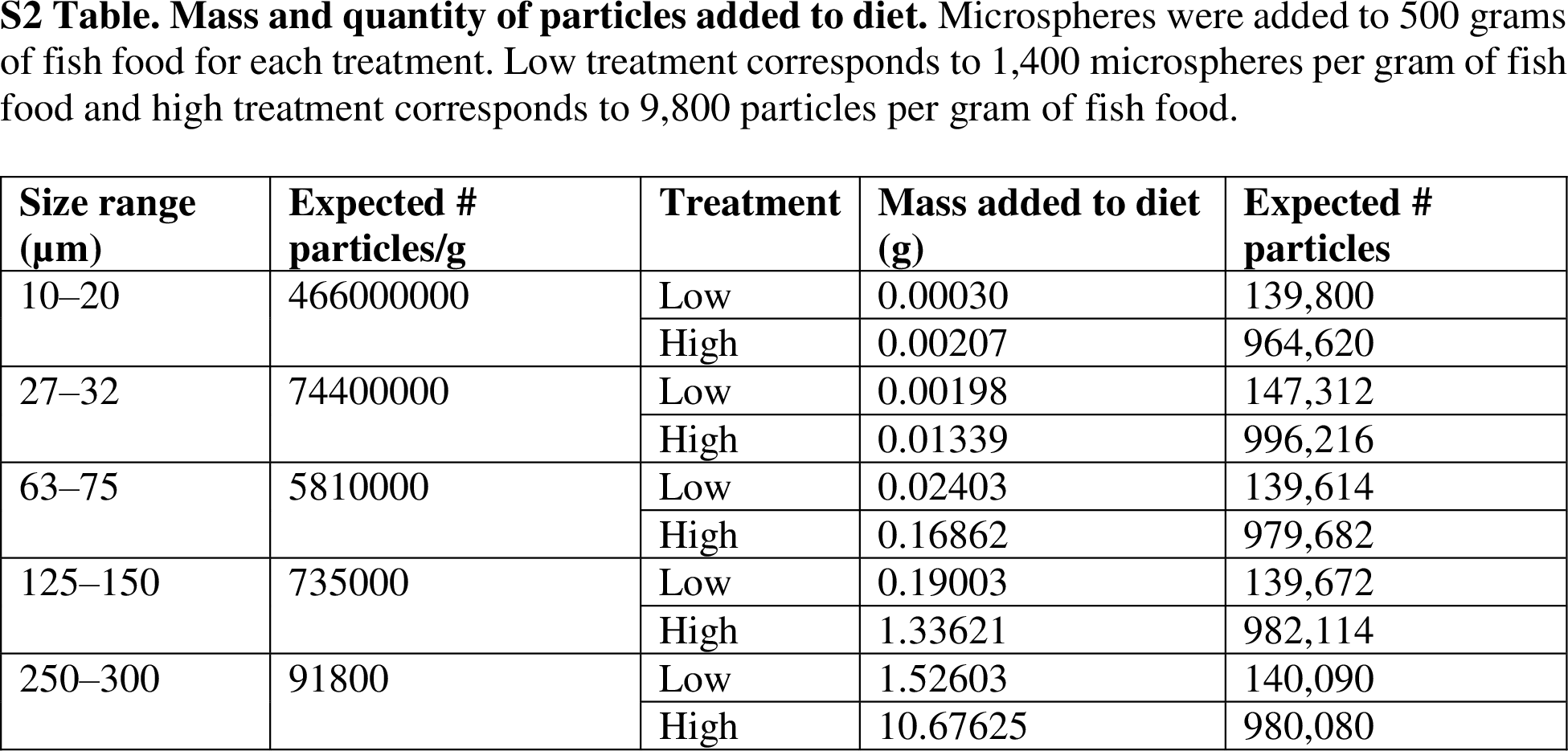

Supplement: S2 Table — Microspheres were added to 500 grams of fish food for each treatment. Low treatment corresponds to 1,400 microspheres per gram of fish food and high treatment corresponds to 9,800 particles per gram of fish food. (TIF) [file pone.0239128.s003.tif]

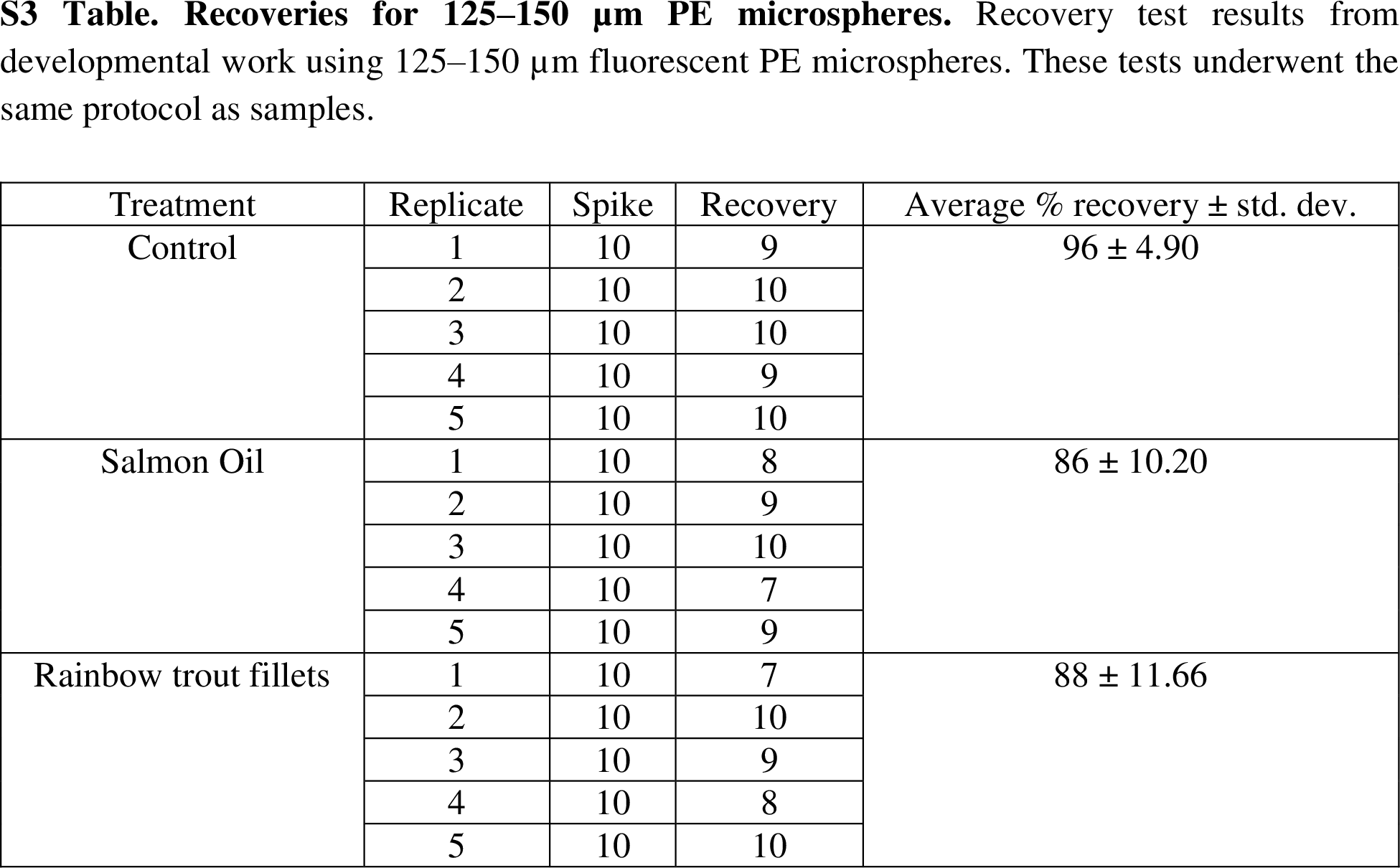

Supplement: S3 Table — Recovery test results from developmental work using 125–150 μm fluorescent PE microspheres. These tests underwent the same protocol as samples. (TIF) [file pone.0239128.s004.tif]

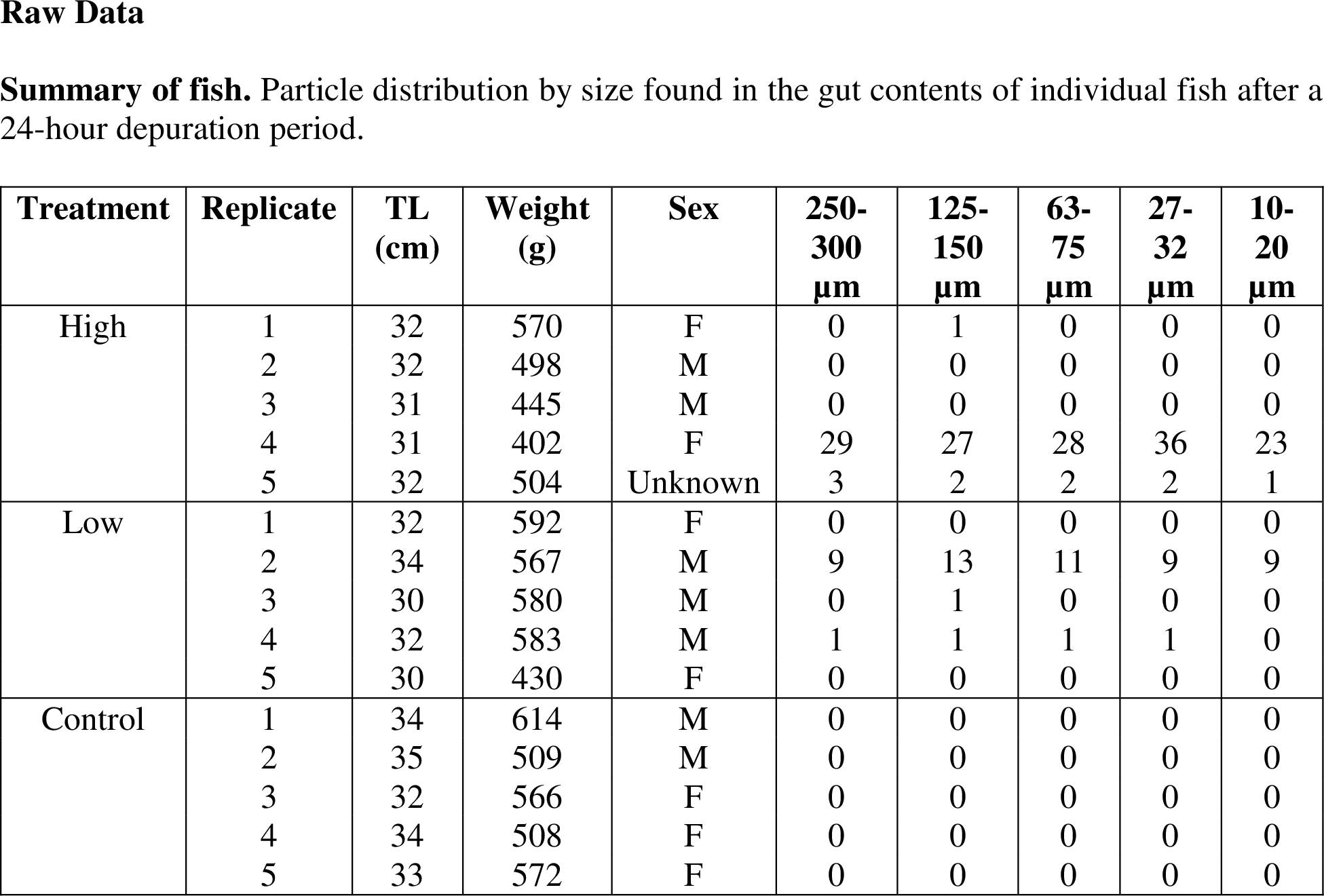

Supplement: S1 Raw data — Particle distribution by size found in the gut contents of individual fish after a 24-hour depuration period. (TIF) [file pone.0239128.s005.tif]
